# Seamless Human–Computer Interaction Enabled by Wearable Biointerfaces and Intelligent Systems

**DOI:** 10.3390/biomimetics11060368

**Published:** 2026-05-26

**Authors:** Huiyu Wei, Jiangbo Hua, Yongchang Jiang, Wenkai Zhu, Wen Cheng, Yi Shi, Lijia Pan

**Affiliations:** 1 Collaborative Innovation Center of Advanced Microstructures, Jiangsu Provincial Key Laboratory of Photonic and Electronic Materials, School of Electronic Science and Engineering, Nanjing University, Nanjing 210093, China; 2 School of Integrated Circuits, Nanjing University, Suzhou 215163, China

**Keywords:** wearable computing, human–computer interaction, multimodal sensing, closed-loop feedback, intelligent systems, flexible electronics

## Abstract

Human–computer interaction (HCI) is central to wearable technology; however, traditional interaction methods face constraints from environmental noise, privacy risks, and operational inconveniences. With the convergence of flexible electronics and artificial intelligence, smart wearable systems equipped with biomimetic biointerfaces are evolving into “external organs” that augment human capabilities, establishing a new paradigm for natural and intelligent interaction. This narrative review provides a comprehensive overview of the research progress in seamless HCI driven by wearable biointerfaces and intelligent systems. From the input perspective, we elucidate how high-fidelity physiological and motion signals are captured through biocompatible electronic skins, and subsequently decoded via intelligent algorithms capable of robust noise decoupling, cross-user generalization, and multimodal data fusion, while emphasizing algorithmic trustworthiness including privacy and interpretability. From the output perspective, we explore adaptive closed-loop feedback mechanisms, spanning both non-visual multi-sensory rendering and biomimetic actuation-based physical interventions. Finally, we discuss key engineering and algorithmic bottlenecks—such as material durability, internal latency, system integration, and trustworthiness—offering future perspectives for the development of next-generation personalized and immersive HCI systems.

## 1. Introduction

In recent years, as key technologies such as micro-nano processing [1], flexible sensors [2], self-powered bioelectronics [3,4], wireless communication [5], and artificial intelligence (AI) [6] in wearable systems have continued to develop, wearable devices have moved from the early proof-of-concept stage to widespread large-scale use. They have demonstrated significant value in fields such as clinical medical assistance [7,8,9], sports and health monitoring [10,11] and immersive entertainment [12]. Compared to traditional portable electronic products like mobile phones, wearable devices are primarily characterized by their long-term attachment or integration onto the human body surface. They can continuously acquire individual physiological states and behavioral information, gradually evolving functionally into “external organs” that assist humans in perception, decision-making, and execution [13]. However, for such human augmentation technologies to function effectively in complex real-world scenarios, it is essential to enable efficient, reliable, and natural information exchange between humans and devices, which means building a highly fluent, highly accurate, and highly intelligent HCI mechanism.

For a long time, traditional wearable devices have mainly relied on interaction methods such as voice commands, physical buttons, or touch screen taps, which have achieved relatively mature applications in wearable consumer electronics like smartwatches [14] and smart glasses [15]. However, in practical use, these interaction methods still face multiple challenges. Voice interaction is susceptible to environmental noise interference and poses privacy leakage risks [16], while touch and button operations often occupy both hands and interrupt current task workflows, causing inconvenience for applications in high-load scenarios [17]. These problems make it difficult for traditional interaction methods to support a truly seamless HCI experience. To this end, current wearable systems are gradually shifting towards flexible sensing technology. Skin-attached or woven flexible sensors continuously perceive the human body state, including physiological and motion states, and convert them into control signals, thereby achieving a more natural and implicit HCI experience that correlates deeply with human body state [18]. The human state information acquired by wearable systems can be used independently for HCI control. For example, the electroencephalogram (EEG)-based brain–computer interfaces (BCI) can be used to guide the auxiliary movement of an exoskeleton robot [19]. It can also be used collaboratively through multi-modal fusion to support more complex and sophisticated interactive tasks, such as human activity recognition by fusing signals from accelerometers and gyroscopes [20]. Utilizing human state information as an input source provides a practical solution to overcome the limitations of traditional interaction methods. This solution has outstanding potential in reducing user cognitive load, enhancing the motor abilities of people with disabilities, and improving the immersion of virtual reality (VR) or augmented reality (AR) systems [21]. However, it also places higher demands on sensor biocompatibility, long-term signal stability, and cross-user generalization capabilities [22,23].

At the output level, devices typically provide feedback or intervention to the human body in two main ways. The first is informational feedback, such as tactile [24], auditory [25], or thermal sensations [26] based on sensory channels. This type of feedback has the advantages of strong privacy, low environmental interference, and high immersion. It can provide timely prompts when the user’s vision is limited (especially helpful for the visually impaired) or when attention is focused on other tasks, making the interaction experience more natural and efficient [27]. The second is actuation-based feedback, where the device directly implements operational interventions on the human body based on real-time sensed data. For instance, Li et al. [28] introduced a real-time monitoring mechanism for surface electromyography (sEMG) in lower-limb exoskeletons, enabling dynamic feedback to the human body at the macroscopic mechanical level based on sEMG. Actuation-based feedback features rapid response and precise intervention, playing an important role in medical-related scenarios such as neuromodulation [29], as well as in sports assistance scenarios [30]. After the feedback occurs, the user dynamically adjusts their own state or behavior based on the device’s output and transmits the updated information back to the device. This forms a continuously running closed-loop HCI process of “sensor perception—algorithm decision-making—actuator feedback—human re-response,” endowing the system with higher levels of intelligence and adaptability, and bringing immense promise for advancing the personalized application of wearable devices [31].

Furthermore, to build intelligent HCI systems that support seamless experiences, related research is continuously advancing in both flexible hardware interfaces and intelligent analysis algorithms. On the one hand, the development of biocompatible materials, flexible stretchable structures, and multimodal sensing technologies has significantly enhanced the signal fidelity, wearing comfort, and dynamic conformability of wearable devices at the hardware level; on the other hand, the introduction of multimodal data fusion strategies, machine learning (ML) architectures, and personalized AI technologies targeting cross-user generalization, on-device learning, and interpretability has provided entirely new solutions for real-time and precise decoding of complex human intentions. The synergistic evolution of these hardware and software technologies collectively drives the development of wearable HCI systems toward high robustness and deep personalization, making the HCI experience more seamless and natural.

Based on the above background, this narrative review provides a comprehensive overview of the research progress in technologies related to wearable biointerfaces and intelligent systems. In Section 2, we focus on cutting-edge hardware and software technologies designed to enhance interaction seamlessness and intent decoding robustness, delving from biocompatible interfaces and multimodal signal sensing at the hardware level to data fusion strategies at the software level, as well as intelligent algorithms addressing personalization and privacy needs. In Section 3, we review the evolution of wearable feedback and actuation technologies within closed-loop interaction systems, covering multi-sensory informational feedback (e.g., haptic and olfactory feedback) and biomimetic actuation-based physical interventions (e.g., neuromodulation and locomotion assistance). Finally, in Section 4, we analyze and summarize the key technical bottlenecks currently confronting the field, followed by a discussion and outlook on potential future development directions and application prospects.

To ensure a rigorous and comprehensive synthesis of the field, a structured literature search was conducted across major academic databases, primarily Web of Science (WoS), PubMed and IEEE Xplore. The search predominantly covered the period from 2017 to 2026 to capture the most recent technological advancements. Specifically, we utilized granular keyword combinations bridging wearable platforms with exact sensing, decoding, or feedback modalities, rather than solely relying on the broad macro-concept of HCI. Representative search strings included [“flexible sensor” OR “wearable device”] AND [“machine learning” OR “deep learning” OR “sensor fusion”] for the input/processing end, and [“wearable” OR “soft robotics”] AND [“haptic feedback” OR “exoskeleton” OR “electrical stimulation”] for the output end. Additionally, targeted material queries such as [“MXene” OR “hydrogel” OR “paper-based”] were incorporated to capture the latest fundamental advancements in biomimetic interfaces.

As this manuscript is presented as a narrative review, the inclusion criteria focused on peer-reviewed studies that:

- demonstrated seamless HCI mechanisms, either utilizing flexible biosensors for human state input or employing multimodal/actuation strategies for closed-loop feedback;
- integrated ML architectures or multi-sensor data fusion strategies to achieve robust noise decoupling and precise intention decoding;
- deployed innovative sensory feedback setups or biomimetic actuation interfaces to construct an adaptive, real-time “sensing-decision-response” loop.

**Figure 1:**
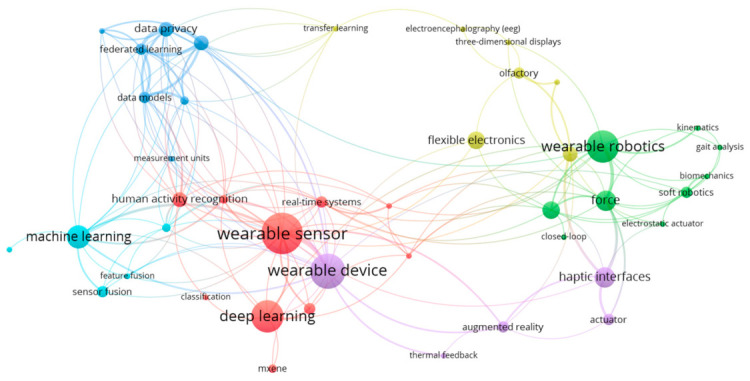
Keyword co-occurrence network visualization of the literature cited in this review (Generated by VOSviewer 1.6.20).

Redundant or low-relevance entries were excluded through manual screening, ensuring that the final selection of literature concisely and firmly supports the core theme of next-generation, seamless HCI systems (Figure 1).

## 2. Toward Seamless Physiological and Motion-Based Inputs

Establishing efficient and stable input channels to acquire human state information is the first step toward realizing seamless HCI. However, during dynamic contact with the skin, flexible sensors inevitably encounter challenges such as motion artifacts, electrode displacement, and individual physiological differences. These factors compromise the fidelity of the raw signals, interfere with back-end algorithmic decision-making, and subsequently limit the feedback precision of the device. Therefore, this section reviews the strategies to address these challenges from three perspectives, aiming to elucidate how input data can be seamlessly transformed into precise information. First, we explore how emerging flexible materials achieve conformal contact with the skin via modulus matching, thereby ensuring the authenticity and reliability of the sensor data. Second, we elaborate on how ML further enhances the interpretability of sensor data through denoising and classification algorithms, and how it achieves personalized calibration via adaptive models. Thirdly, we analyze the practical challenges faced by algorithms applied in wearable devices, including datasets, evaluation metrics, privacy issues, and interpretability; Finally, we analyze how multimodal fusion overcomes the limitations of single-modal perception to ultimately facilitate more rational decision-making.

### 2.1. Biocompatible Interfaces for Mechanical Match

#### 2.1.1. Dynamic Conformable Interfaces for Mechanical Match

**Figure 2:**
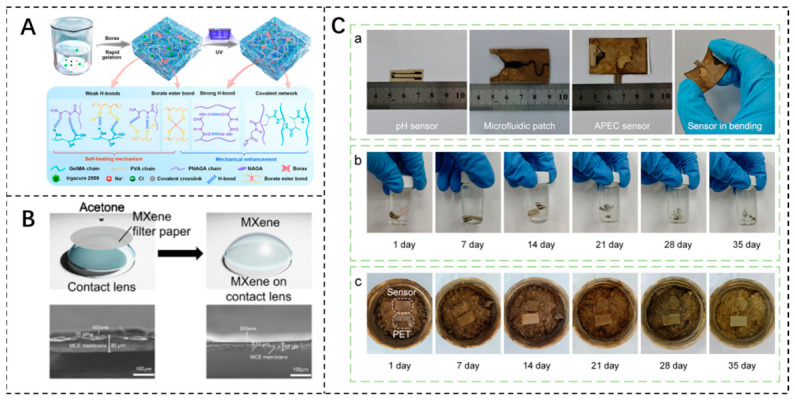
(**A**) Schematic of the fabrication process, internal interactions, and synthesis mechanism of the proposed GelMA-based hydrogel. Reprinted from Ref. [35] under the terms of the CC BY license. (Copyright © 2025 The Authors. Advanced Science published by Wiley-VCH GmbH). (**B**) Schematic of transferring MXene onto a soft contact lens, and cross-sectional SEM images of the MXene film and the acetone-treated MXene film. Reprinted from Ref. [36] under the terms of the CC BY licene. (Copyright © 2025 The authors. Small Science published by Wiley-VCH GmbH). (**C**) (**a**) Photographs and dimensions of the paper-based pH sensor, microfluidic patch, and APEC sensor, and the APEC sensor under bending. (**b**) Biodegradation of an APEC sensor and a polyethylene terephthalate (PET) control film in DI water over 35 days. (**c**) Biodegradation of an APEC sensor and a PET control film in soil over 35 days. Reprinted with the permission from Ref. [37]. (Copyright © 2025, American Chemical Society).

As a natural biological interface, human skin possesses multimodal characteristics such as a low Young’s modulus, high stretchability, and self-healing capabilities, enabling it to perfectly conform to various complex human movements. Therefore, to achieve truly seamless HCI, the information acquisition end of wearable systems must highly mimic natural skin in terms of physical and mechanical properties. However, when subjected to dynamic deformations such as human joint bending and muscle contraction, traditional rigid sensors are prone to interfacial slip or detachment. This mechanical mismatch not only disrupts the user’s unobtrusive experience during interaction but is also the root cause of introducing motion artifacts and causing sensor data distortion [32]. Therefore, utilizing flexible materials to eliminate the modulus disparity between the device and the skin, and constructing an intelligent biomimetic interface capable of dynamic conformal contact (Figure 2), is the underlying core to overcoming physical interference and ensuring high fidelity at the interactive input end.

As a typical biomimetic material, hydrogels possess a hydrophilic three-dimensional cross-linked network highly similar to the matrix structure of human soft tissues. Their intrinsic Young’s modulus naturally matches that of the skin, making them an ideal platform for constructing imperceptible flexible interactive interfaces [33]. In interaction intent recognition scenarios, researchers have utilized flexible hydrogel optical fibers to fabricate wearable curvature sensors, successfully achieving the precise extraction of control commands from large-curvature human movements (such as elbows and knees) [34]. These biomimetic optical fibers can maintain conformal contact with human skin even under extreme deformation (at a high curvature of 139.84 m^−1^), overcoming the defects of traditional rigid silica optical fibers, which are easily damaged and unable to conform to natural human movements. This seamless mechanical fit enables the system to unobtrusively and stably perceive human dynamics, thereby broadening the mechanical boundaries of implicit interactive inputs.

However, in all-weather daily interactions, interfacial materials often face mechanical fatigue and physical damage under long-term and repeated large-strain stretching, leading to the drift or even interruption of interactive signals. The key to solving this problem lies in endowing the interfacial materials with self-healing capabilities, enabling them to autonomously recover their structure and functionality after damage. Due to the integration of reversible dynamic chemical bonds, self-healing hydrogels can autonomously recover their structure and sensing functionality after physical damage, becoming an ideal choice for eliminating interfacial mechanical fatigue [38]. Although traditional self-healing hydrogels generally face the challenge of insufficient mechanical strength [39], recent studies have successfully broken through this limitation by constructing intelligent multi-level energy dissipation mechanisms. For instance, Li et al. [35] designed a gelatin methacryloyl (GelMA)-based biomimetic hydrogel (Figure 2A) that utilizes a large number of internal reversible dynamic bonds as sacrificial bonds to dissipate external deformation energy, synergistically achieving a high self-healing efficiency (86%) and excellent stretchability (approximately 160%). This biomimetic interface design, possessing both physical toughness and self-healing capability, effectively extends the interactive lifespan of wearable systems, ensuring the robustness and seamlessness of human–computer connections in long-term wearing scenarios.

In addition to intrinsically flexible hydrogels, two-dimensional (2D) transition metal carbides/nitrides (MXenes), which possess electrical conductivity comparable to metals, are also important materials for fabricating high-fidelity interactive interfaces. Although the high Young’s modulus of MXene monolayers inherently limits their ability to achieve direct, tight conformal contact with the skin [40], strategies involving structural biomimetics or matrix compositing can be utilized in the design of interaction systems to optimize their mechanical compliance. For example, by constructing a three-dimensional (3D) porous framework that mimics natural biological sponges [41], or by embedding MXenes into flexible polymer and fabric matrices [42], devices can be endowed with excellent mechanical buffering properties and toughness, thereby still achieving seamless physical integration with curved skin surfaces during human movement. Although, compared to hydrogels, MXenes face the problem of being prone to oxidative degradation in open daily environments, by leveraging their abundant surface functional groups to introduce structural protection, an optimal balance among mechanical strength, flexibility, and long-term environmental tolerance can also be achieved [43]. This multi-dimensional optimization, which combines superior electrical transport with physical compliance, ensures that the input end can acquire pure, low-latency signals in complex interaction scenarios.

#### 2.1.2. Ensuring Electrical Fidelity Against Interferences

Because electrophysiological signals are extremely weak and accompanied by complex micro-movements of deep muscles, the high-fidelity extraction of signals remains challenging even if the interfacial materials have physically achieved conformal contact. Furthermore, in practical wearable HCI scenarios, complex external electromagnetic radiation, temperature and humidity fluctuations, as well as the electrical hysteresis of the materials themselves, will introduce a massive amount of multi-source noise, resulting in the distortion and delay of interactive commands. Therefore, starting from the intrinsic electrical properties of the materials, simulating the high signal-to-noise ratio and anti-interference mechanisms of biological nerves to construct a biomimetic electrical interface with strong anti-interference capabilities is another key dimension to ensure the seamless transmission of information flow in HCI.

The unique layered framework of MXenes can induce multiple internal reflections and dissipation of electromagnetic waves, thereby providing highly efficient shielding protection for weak bioelectrical signals and preventing external stray electromagnetic fields from disrupting the “human–computer” communication channel. This demonstrates their immense potential in constructing smart interactive interfaces with anti-electromagnetic interference capabilities [40]. For example, in eye-tracking and electrooculogram (EOG) interaction tasks, researchers have integrated a Ti3C2Tx MXene film onto the surface of a commercial contact lens (Figure 2B) [36]. Utilizing the shielding properties of the material, this design effectively isolates the electrical interference caused by external high-frequency radiation on EOG interactive signals in daily environments (achieving an electromagnetic shielding rate of 85%), thereby ensuring the high precision and coherence of hands-free control commands. Furthermore, through hybridization with biomass materials (such as cellulose nanofibers), MXenes can further reduce thermoelectric signal interference caused by external temperature fluctuations (with the Seebeck coefficient as low as −3.83 μV K^−1^). This enables wearable interactive systems to adapt to diverse and dynamically changing real-world daily environments, maintaining the long-term stability of seamless human–computer communication [44].

In addition to external interference, physical interference that may occur during the repeated stretching and recovery of materials—namely, electrical hysteresis—is another important cause of signal distortion. In flexible sensors, if the internal conductive network cannot completely and synchronously recover after the material undergoes large strain, it will lead to inconsistent electrical signal outputs under the same deformation. This temporal lag contradicts the immediacy required by seamless interaction. To eliminate the noise caused by the viscoelasticity of the material, researchers improve electrical hysteresis by designing rapidly reversible dynamic conductive networks. For instance, introducing a small-molecule glycerol regulation mechanism into the hydrogel conductive network with abundant dynamic bonds enables the system to achieve an extremely low hysteresis rate (0.64%) [45]. This means that the resistance changes in the sensor can strictly and synchronously follow the real-time movement of the human body, fundamentally eliminating the dynamic hysteresis in signal transmission and providing a high-fidelity, zero-latency control source of motor intention for back-end algorithms. Although such hydrogel-based sensors still have room for further optimization in output power [46], their biomimetic logic in eliminating system time differences and advancing instantaneous closed-loop interaction provides crucial support for next-generation highly sensitive human–computer interfaces.

#### 2.1.3. High Biocompatibility for Long-Term Wearing

When designing a seamless HCI interface, it is necessary to consider not only the mechanical and electrical properties of the materials but also ensure that the device has long-term biocompatibility. Although traditional dense flexible polymer substrates possess a certain degree of flexibility, their lack of breathability makes them prone to causing the localized accumulation of sweat and heat during long-term skin-attached use. This can not only trigger the risk of inflammation and allergies but also lead to the peeling of electrodes or interfacial impedance fluctuations due to changes in the interfacial microenvironment (e.g., a sharp increase in local humidity), thereby forcing the interruption of the interactive process [47]. Therefore, to ensure comfort during the seamless interaction process, it is imperative to develop biomimetic breathable materials with excellent microenvironment regulation capabilities.

As an emerging green platform, paper-based electronics provide an ideal solution for mimicking the microenvironment regulation mechanism of the skin. Paper is primarily composed of natural cellulose fibers and features characteristics such as being lightweight, low-cost, and having excellent biodegradability. Distinct from the dense 2D layered structure of MXenes, paper, which is predominantly made of natural cellulose, possesses a unique 3D porous network structure. This physical structure biomimetically matches the breathable pores of the biological epidermis to a high degree, endowing paper-based devices with superior breathability and sweat dissipation capabilities, which offers significant advantages in maintaining the homeostasis of the skin microenvironment and avoiding rejection reactions [48]. Furthermore, by introducing origami structures [49] or compositing with nanomaterials [50], the inherent limitation of low stretchability in paper-based materials can be overcome, enabling them to serve as comfortable and invisible flexible sensors to continuously capture behavioral interaction intentions.

Furthermore, while exhibiting excellent biocompatibility, paper-based materials also possess fully degradable characteristics, and their manufacturing processes are typically simpler and non-toxic, effectively avoiding the generation of electronic waste. This allows paper-based wearable devices to simultaneously satisfy both human-friendly and environmentally friendly requirements [51]. For instance, Li et al. [37] developed an all-paper-based degradable electrochemical sensor that achieved highly sensitive monitoring of human sweat pH with a high sensitivity of up to 46.01 mV pH^−1^. More notably, the fabrication of this device requires neither masks nor organic solvents, and the device itself is completely biodegradable (Figure 2C). This non-toxic, fully degradable, and environmentally friendly characteristic perfectly aligns with the development requirements for future wearable devices to achieve large-scale and normalized integration as human “external organs.”

However, the exceptional hydrophilicity of paper-based materials, while endowing them with superior breathability, also exposes them to challenges such as structural softening and electrical signal drift in high-humidity interactive scenarios, such as post-exercise sweating [51]. Therefore, in future research, in addition to exploring composites with other functional materials to enhance mechanical toughness, it is necessary to utilize localized hydrophobic modification or semi-permeable membrane encapsulation technologies. This will improve water resistance while maintaining high biomimetic breathability, thereby ensuring the continuity and robustness of human–computer connections in complex and dynamic scenarios.

To provide a comprehensive and critical review of these technical advancements, Table 1 categorizes and compares representative works based on their critical indicators.

### 2.2. Signal Fidelity via Algorithmic Noise Decoupling

As a prominent algorithmic approach, ML enhances the real-time data processing performance of software algorithms through a systematic pipeline of “data preprocessing—feature extraction—model training—result evaluation,” thereby gaining widespread attention from researchers. On the one hand, relying on its robust nonlinear feature extraction capabilities, ML can extract and reconstruct weak, authentic physiological signals from high-dimensional and noisy backgrounds. This reduces the impact of motion artifacts at the software level and improves signal fidelity. On the other hand, ML models support continuous baseline calibration for the specific data features of different users through few-shot learning or adaptive fine-tuning. This mitigates the impact of individual differences and enables accurate, personalized analysis and health assessment [21]. Currently, various ML algorithmic architectures have been integrated into the data processing workflows of wearable sensors (see Table 2) to ensure that the system can still output highly reliable and interpretable interactive commands when facing complex interference.

When wearable devices realize seamless HCI, the fidelity of input signals is often compromised by complex environments. During long-term daily wear, vigorous limb movements inevitably introduce motion artifacts, while the electrophysiological activities of adjacent muscles or organs lead to crosstalk and non-linear distortion of target intent signals [58]. Traditional linear filtering algorithms face an inherent contradiction when processing such multi-source, non-stationary noise: if the filtering threshold is set too wide, the interference cannot be effectively filtered out; if set too narrow, it easily leads to the loss of key physiological features, subsequently triggering false execution or latency of interaction commands [59]. From a biomimetic perspective, the human central nervous system possesses an efficient “sensory gating” mechanism that can subconsciously filter out background somatosensory noise, such as respiration and gaits, to precisely extract core external stimulus information [60]. To reproduce this mechanism on wearable hardware, intelligent algorithms have been widely introduced. Their core task is to construct a digital, adaptive gating system at the software level, achieving noise decoupling without disrupting the original intentions, thereby providing a pure command source for seamless closed-loop interaction [61].

To achieve this decoupling at the computation-constrained wearable edge, architectures combining mathematical signal separation and traditional ML classifiers have been proven to be an efficient approach. Such methods do not require reconstructing perfectly pure waveforms at the front end; instead, they directly construct nonlinear decision boundaries in a high-dimensional feature space. For example, targeting the complex physiological crosstalk caused by EOG and ECG in EEG interactions, Yu et al. [62] proposed an identification framework based on blind source separation (BSS). They eliminated feature artifacts through a Second-Order Blind Identification (SOBI) algorithm and subsequently applied RF to extract multi-dimensional time-frequency features, achieving a classification accuracy of up to 85.2% for pre-fall anticipation on a single occipital channel (O2). Because it does not rely on dense tensor operations, this strategy exhibits excellent computational efficiency, making it an ideal solution for constructing zero-latency seamless human–computer interfaces. However, the limitation of this mechanism lies in its heavy reliance on hand-crafted statistical features; when the system faces highly random and intense mechanical vibration noise in real-world scenarios (such as vigorous movements like running), the feature extraction effectiveness of such shallow models often fails to meet practical requirements [63].

In contrast, deep learning models with deep network architectures, such as CNNs, exhibit intrinsic nonlinear feature extraction and adaptive decoupling capabilities for spatiotemporal patterns. For instance, Chen et al. [64] proposed a noise-tolerant deep learning-enhanced wearable system. They constructed a composite dataset containing various mechanical vibrations and human motion artifacts to train a customized CNNs, enabling the network to autonomously learn the essential differences in spatial topology between the target signals and background noise. Ultimately, a high recognition accuracy of 90.8% for gesture control intentions was maintained even when the signal-to-noise ratio dropped to a low level of −10 dB. Furthermore, the translation invariance of CNNs effectively reduces uncontrollable interference caused by electrode slippage during long-term wear [56]. However, obtaining such high-fidelity denoising performance often requires a compromise in computational and temporal resources, as the dense floating-point operations of deep networks increase the energy consumption burden on edge devices while simultaneously introducing a high inference latency [65].

### 2.3. Multimodal Sensing and Data Fusion

#### 2.3.1. Multimodal Signal Sensing and Physical Integration

Although single-modal sensing mechanisms have demonstrated favorable performance in specific tasks, in real, complex, and dynamically changing practical application scenarios, relying solely on a single or multiple homogeneous sensors often struggles to support stable and natural HCI. This is because this approach typically faces issues such as limited information dimensions, sensitivity to environmental noise, and insufficient interactive semantic parsing capabilities, thereby limiting the reliability and scalability of the system. To break through this limitation, wearable systems have introduced multimodal fusion sensing strategies, aiming to provide interactive inputs with high fidelity and strong robustness for the underlying algorithms [66].

Among multimodal fusion schemes, the synergistic application of inertial measurement units (IMUs) and electrophysiological signals (such as sEMG) is particularly typical. These two types of signals possess a natural complementarity in time scales and physical mechanisms: IMUs can provide robust and continuous body kinematics information, while electrophysiological signals reflect the neuromuscular activation processes that precede or accompany observable movements. By fusing these two types of signals, the system can not only perceive the outcomes of movements but also pre-emptively infer the user’s motor intentions. Currently, IMU–sEMG fusion has achieved multiple breakthroughs in complex intent decoding tasks. For instance, Yu et al. [67] combined kernel principal component analysis (KPCA) and SVM to jointly model the lower limbs, achieving high accuracies of 95.35% offline and 93.3% online in gait intent recognition. Xu et al. utilized a two-stream CNNs to fuse the energy kernel phase diagram of sEMG and the amplitude images of IMU, achieving an average recognition accuracy of 95.78% across 6 gestures, which is significantly superior to single-modal sensing schemes. Furthermore, in addition to discrete intent recognition, this fusion strategy has also demonstrated immense value in continuous human–computer collaborative interactions; for example, targeting populations with upper limb impairments, the IMU–sEMG fusion strategy has been proven to enable precise hands-free control of external assistive devices (such as robotic arms) [68].

Notably, multimodal fusion strategies have also been widely investigated in the field of physiological state monitoring (such as fusing ECG and PPG signals for respiratory estimation [69]). However, these physiological fusion strategies are primarily designed for long-term, static vital signs monitoring. Although they exhibit excellent performance in monitoring accuracy, they struggle to satisfy the stringent requirements for instantaneous responses in seamless HCI (such as the dynamic following of exoskeletons), and their potential for directly driving low-latency seamless interaction remains limited.

#### 2.3.2. ML-Enabled Data Fusion Strategies

**Figure 3:**
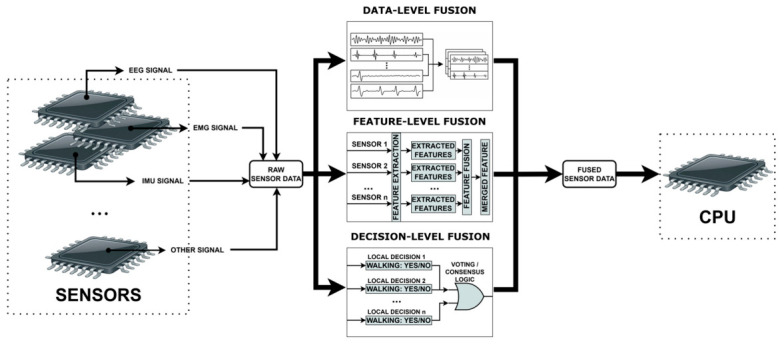
After multimodal data is acquired by the sensors, intelligent algorithms such as AI or ML are employed to implement three distinct fusion strategies: data-level fusion, feature-level fusion, and decision-level fusion. Upon completion of the fusion process, the data is output to the central processing unit (CPU) for subsequent decision-making.

After acquiring sensor data, there are typically three fusion methods (see Figure 3): data-level fusion, feature-level fusion, and decision-level fusion, with each stage affecting interaction performance differently [70].

Data-level fusion is the lowest-level fusion strategy, which directly concatenates and integrates data at the raw sensor signal level, with the advantage of maximizing the retention of underlying physical information. Kolanowski et al. [71] proposed using the feedback layer of an Elman Recurrent Neural Network (RNN) to explicitly model temporal dynamics, avoiding complex preprocessing or explicit alignment, and achieved high-precision estimation of Euler angles (with errors for the three angles ranging between 1.4° and 2.1°) by fusing raw data from an accelerometer, gyroscope, and magnetometer. However, from the perspective of practical deployment on wearable devices, this strategy possesses significant limitations: it demands high temporal synchronization of heterogeneous signals. Directly processing high-frequency, high-dimensional raw multimodal data on computation-constrained microcontrollers not only incurs a massive memory footprint but is also prone to causing inference latency due to dynamic noise, making it difficult to satisfy the instantaneous response requirements needed for seamless interaction [72].

In contrast, feature-level fusion is a data processing strategy that synthesizes and optimizes extracted feature representations in multi-sensor systems. Compared to direct concatenation at the raw data level, it first extracts high-order features from individual sensors before performing concatenation and optimization, which enables it to effectively filter underlying dynamic noise and substantially compress data dimensions. For instance, in complex human activity recognition (HAR) tasks, Zeng et al. [73] proposed a residual multi-feature fusion shrinkage network, utilizing a channel attention mechanism to automatically select the most discriminative features, achieving high recognition accuracies across multiple public datasets (e.g., 98.35% on WISDM and 98.13% on UCI-HAR). The UC Fusion method by Liu et al. [74] innovatively separated the unique and common features of each sensor before fusion, likewise achieving an excellent classification performance of 98.85% on WISDM. Because feature-level fusion achieves an optimal balance between model parameter efficiency and feature representation capability, it effectively alleviates the computational pressure on edge devices, making it an ideal architecture to support low-latency seamless interaction [75].

Distinct from data-level fusion and feature-level fusion, decision-level fusion (also known as late fusion) delays information integration until after each modality generates an independent prediction, systematically combining hypotheses from multiple classifiers to produce the final output. The primary advantage of this strategy lies in its ability to better preserve the independence of each modality, effectively avoiding feature conflict issues caused by the direct concatenation of multi-source data [76]. Benefiting from these advantages, decision-level fusion strategies have also become an important means of processing complex HAR data. For example, Yadav et al. [77] proposed a two-stream decision-level fusion system that fuses visual spatial features and inertial temporal features at the final classification stage, achieving high recognition accuracies ranging from 95.9% to 98.7% across multiple multimodal HAR datasets (such as UTDMHAD, Berkeley-MHAD, and C-MHAD). In seamless interaction scenarios, the greatest engineering advantage of decision-level fusion lies in its exceptional fault tolerance: even if some flexible sensors fail due to sweat interference or electrode peeling, the system can still rely on the predictions of other modalities to “vote,” thereby maintaining basic interactive functionality [78].

### 2.4. Trustworthy Algorithms for Seamless HCI

#### 2.4.1. Datasets and System Evaluation Metrics

In deep learning-driven wearable HCI research, high-quality datasets serve as the underlying cornerstone that determines the generalization capability and robustness of algorithms. Unlike the fields of computer vision or natural language processing, which possess ultra-large-scale and highly standardized open-source datasets (such as ImageNet), the field of wearable biological interfaces (encompassing multimodal physiological signals such as sEMG, EEG, and IMUs) still significantly lacks unified, large-scale open-source benchmark datasets [79]. Although the academic community has recently open-sourced some comprehensive and representative multimodal physiological feature databases—for instance, the large-scale hand biomechanics dataset, BHaM, released by Diaz et al. [80], which provides rich labels including sEMG, high-precision kinematics, and dynamics—most of these high-quality multimodal datasets must still be collected in highly lab-controlled environments, where subjects typically execute preset, regular movements under specific constraints. Furthermore, restricted by collection costs, the sample size for high-fidelity time-series signals is often limited to a few dozen individuals (for example, the complete biomechanical dataset of BHaM includes only 30 subjects).

This objective data distribution discrepancy, known as a domain shift, between lab-constrained data and the natural real world makes it difficult for intelligent algorithms pre-trained on such data to cope with the highly unstructured motion artifacts induced by sweating, electrode shifts, and irregular daily environmental interactions in long-term wearable scenarios [81]. Moreover, the fragmentation and lack of standardization across different research institutions regarding sensor spatial layouts, hardware sampling rates, and data annotation formats severely hinder the fair performance comparison and cross-study replication of various algorithms [79]. Consequently, constructing large-scale wearable multimodal physiological datasets that cover long-term activities of daily living (ADL), incorporate multi-source environmental noise, and establish standardized access frameworks can not only help resolve current generalization issues but also propel the underlying algorithms of wearable systems toward highly generalizable foundation models [82].

In addition, regarding the construction of system evaluation frameworks, most current wearable biological signal classification studies predominantly rely on static metrics from the traditional ML domain, such as accuracy, precision, and F1-score. Although these metrics can intuitively reflect the static fitting capability of models on pre-split test sets, they decouple from the physical resource constraints of wearable edge hardware as well as the time sensitivity of closed-loop HCI. This necessitates that the evaluation criteria for wearable intelligent algorithms also incorporate engineering indicators such as inference latency, energy efficiency, and long-term robustness [83].

#### 2.4.2. Cross-User Generalization and On-Device Learning

When wearable devices are deployed across users, the generalization capability of pre-trained models often significantly degrades due to objective differences in individual physiological structures, skin impedance, and movement habits [84]. To overcome this challenge of cross-user generalization, transfer learning based on deep convolutional networks has been widely applied. This technology leverages the general features extracted from source domain models, requiring only a small amount of personalized data from target users to fine-tune the deep network weights, thereby enhancing the model’s cross-user generalization capability [85]. For instance, Ruhrberg Estévez et al. [86] utilized a transfer learning architecture to fine-tune a locomotion intent classification network for ankle exoskeletons, enabling the system to achieve reliable adaptation to new users with a minimal collection of only 10 calibration samples per motion class, achieving a classification accuracy as high as 99.26%. Furthermore, the transfer learning-based gesture recognition strategy proposed by Chen et al. [64] requires only 2 samples per gesture to elevate the recognition accuracy of new users from 51% to over 92%.

However, traditional deep learning models typically require intensive backpropagation and extensive floating-point operations during adaptive calibration. Such iterative update mechanisms impose significant pressure on the computing power and power consumption of standalone wearable devices, making it difficult for the system to perform local retraining; consequently, systems often resort to cloud-based retraining, which entails higher latency and privacy risks [87]. This contradicts the immediacy and security requirements of seamless closed-loop interaction. Therefore, exploring on-device learning mechanisms capable of directly executing model parameter updates locally at the sensor or edge device level to achieve truly secure and immediate personalized calibration has become an inevitable trend in current system design [88].

Although on-device learning provides an ideal framework for cross-user generalization, deploying continuous adaptive updates on resource-constrained microcontrollers (MCUs) or flexible sensor nodes still faces severe challenges. This is because gradient-based updates driven by backpropagation in traditional deep learning incur extremely high memory footprints and power consumption overheads, constituting the primary computational bottleneck for the physical deployment of on-device learning [89]. To break through the limitations imposed by hardware physical resources on on-device learning, research has gradually shifted toward lightweight, non-gradient-dependent computing architectures. Among these, brain-inspired hyperdimensional computing (HDC) maps low-dimensional signals into hyper-high-dimensional binary vectors and utilizes simple bitwise logic operations to replace complex multiply-accumulate operations, thereby drastically reducing the power consumption and computational demands of model updates from the underlying logic [65]. For example, Moin et al. [65] developed an adaptive surface electromyography (sEMG) sensing system integrated with an HDC algorithm, achieving a classification accuracy of 97.12% for 13 gestures under single-trial training. Notably, when confronting dynamic interferences such as physical sensor shifts that lead to performance degradation, the system can execute incremental learning entirely locally on the wearable device, restoring the recognition accuracy by 9.5% without connecting to an external computing platform. This local imperceptible calibration achieved with low computational overhead provides a practical and feasible technological pathway for constructing adaptive seamless interaction systems with long-term robustness.

#### 2.4.3. Privacy and Security Perservation

Wearable interactive systems inevitably collect highly sensitive multimodal physiological data (such as continuous sEMG, ECG, and gait trajectories). Traditional cloud-based centralized training exposes these raw signals, which contain strong biometric signatures, to communication links, raising severe privacy leakage concerns [90]. To protect user privacy without sacrificing model generalization capabilities, federated learning (FL) has emerged as a core paradigm in current system architecture design. The fundamental principle of this mechanism is “keeping data local while models move,” where raw physiological data are strictly restricted to wearable edge devices for local model training, and the device only encrypts and uploads the calculated model gradients or weight updates (model updates) to a cloud server for global parameter aggregation [91]. For instance, Chen et al. [92] developed a federated transfer learning framework (FedHealth) for wearable healthcare and locomotion interactive systems. Without sharing raw user data, this system provides an average accuracy of up to 99.4% for personalized activity recognition solely through cross-device model parameter aggregation and cloud collaboration, achieving a balance between model performance and physical data isolation at the architectural level.

However, merely isolating raw data is not absolutely secure; by analyzing the parameters uploaded by clients, a portion of the user’s physiological features could still potentially be reversely reconstructed. To address this vulnerability, differential privacy (DP) technologies have been further integrated into the federated architecture. By purposefully injecting strictly calibrated mathematical noise into the gradient parameters uploaded locally from devices, DP can theoretically limit the impact of a single data sample on the global model, thereby severing the mapping relationship between parameters and personal features [93]. This theoretical mechanism has been quantitatively validated in specific wearable HCI tasks. For example, Favero et al. [94] proposed a differential privacy federated forest algorithm (Federated Forests with DP) for distributed wearable sensors. By introducing Laplace noise when training decision trees locally on the client side, they limited the impact of individual sensor data points on model parameters, achieving a classification accuracy of approximately 70% under strict privacy protection (higher than the 55% achieved by standalone models trained without federated aggregation). These underlying cryptographic and distributed mechanisms provide an indispensable cornerstone for establishing trustworthy and secure seamless wearable HCI in the future.

#### 2.4.4. Model Interpretability

When constructing seamless wearable HCI systems, algorithmic interpretability is the core to ensuring physical safety and establishing user trust. Current deep learning models used for high-fidelity feature extraction (such as CNNs and LSTMs) are inherently “black-boxes,” whose internal, highly non-linear decision logic remains invisible to humans [95]. In closed-loop HCI scenarios that require high trust (such as exoskeleton control), this lack of decision transparency can pose significant physical and medical risks, leading to severe user distrust in the algorithms. To address this issue, post hoc explanation methods have been widely introduced to dissect the decision-making rationale of pre-trained models. Among these, the game theory-based SHAP (SHapley Additive exPlanations) algorithm can provide a unified explanation of global and local feature importance by calculating the marginal contribution value of each feature to the final model output [96]. For example, in lower-limb exoskeleton terrain classification and parameter estimation, Coser et al. [95] introduced SHAP technology to analyze the black-box decision logic of models such as LSTM and CNN-LSTM. The explanation results confirmed that the model predictions align with human biomechanical principles—specifically, that the foot IMU plays a decisive role in feature recognition, while intuitively revealing that EMG signals and the torso IMU make minimal marginal contributions to the model output. Based on this transparent and trustworthy evaluation rationale, they streamlined the wearable system to include only three IMUs on the foot, lower leg, and thigh, while maintaining a high classification accuracy of 0.94. This demonstrates that the introduction of interpretability techniques possesses dual value in ensuring the safety of HCI tasks and enhancing the trustworthiness of wearable systems.

Beyond post hoc explanations, another paradigm is to construct neural network architectures with intrinsic explanatory capabilities by introducing attention mechanisms. This mechanism adaptively assigns weight vectors to different spatial sensor nodes or time-series segments, rendering the model’s “focus” during feature extraction physically visible. The success of deep learning models in time-series classification tasks relies heavily on such mechanisms capable of capturing long-range dependencies and key features [97]. In the field of sEMG-based interactive control, Lee et al. [98] developed a deep network integrating an attention mechanism to decode the complex motion angles of 14 finger joints based on forearm electromyography. The color map of the attention matrix output by this model clearly highlights the sEMG channels assigned with the highest activation weights; notably, the physical locations of these channels automatically given high weights by the algorithm coincide with the muscle groups actually activated during finger movements. This model architecture, which can be directly and visually validated by human physiological prior knowledge, ensures the transparency of the system’s underlying decision logic, laying a fundamental cornerstone for forging the next generation of safe and trustworthy seamless wearable HCI systems.

## 3. Multimodal Feedback Output Technologies

In the progression toward seamless HCI, sensing and decoding the human body state constitutes only half of the system’s capabilities, while it is equally an indispensable key component for the device to deliver computation results back to the user in an appropriate form and act upon the human body. For a long time, devices in HCI have relied on open-loop feedback, which often faces issues of excessive or insufficient feedback intensity and the inability to dynamically adjust based on the user’s real-time responses. In contrast, closed-loop interaction systems emphasize that upon detecting changes in human physiological states or behaviors, the device can proactively apply feedback or interventions, continuously adjusting the system’s behavior through the human body’s re-response to form a continuous and adaptive interactive process [99]. In wearable devices, closed-loop feedback not only serves for information prompting and guidance but also directly participates in functions such as physiological regulation, neural modulation, and motion assistance, exerting a substantial impact on user behavior and states. In this section, we first introduce various feedback methods acting at the sensory level, and subsequently discuss direct intervention feedback based on actuators, demonstrating the potential of closed-loop designs in realizing seamless HCI across diverse application scenarios.

### 3.1. Sensory Feedback

Vision is one of the primary methods through which humans acquire information from the external world. However, during highly complex tasks or specific pathological conditions, the visual channel may suffer interference or even temporary failure, making it difficult to support continuous, high-density information acquisition. To compensate for the perceptual bottlenecks that may emerge in wearable closed-loop HCI systems, it is particularly necessary to develop feedback technologies based on non-visual sensory channels. According to the differences in sensory channels, this section will introduce emerging multi-channel sensory feedback technologies.

#### 3.1.1. Haptic Feedback

The skin is the largest organ of the human body. By stimulating nociceptors, thermoreceptors, and mechanoreceptors located at different depths within the skin, the human body can achieve tactile perception of the external world, thereby forming a personal understanding of the surrounding environment. The generation of a sense of touch is of vital importance in object recognition, motor control, and the realization of social activities [100]. Compared to other sensory channels, haptic feedback technology has become one of the most highly anticipated research directions in HCI due to its advantages of rapid response, high privacy, and strong accessibility. Currently, wearable haptic devices developed based on this technology widely adopt electromechanical actuators as their feedback actuation mechanisms. This is because they possess advantages such as a wide output frequency band, low static power consumption, strong controllability and ease of integration. Furthermore, wearable haptic devices based on electrical stimulation and thermal sensation have also received extensive research attention. Combined with novel materials such as polymeric and fluidic materials, these devices provide crucial support for achieving seamless interaction [101].

##### Electromechanical Actuator-Based Haptic Devices

Wearable haptic devices based on electromechanical actuators are currently the most successfully commercialized category of wearable haptic devices. They utilize motors, linear actuators, and micro-mechanical structures to generate controllable displacement, pressure, or deformation on the surface of human skin, thereby directly stimulating mechanoreceptors to accomplish haptic feedback [102]. To realize a truly seamless HCI experience, these actuators must not only deliver precise mechanical stimuli but also match the spatiotemporal resolution of human tactile perception while simultaneously mitigating the physical burden on the body.

**Figure 4:**
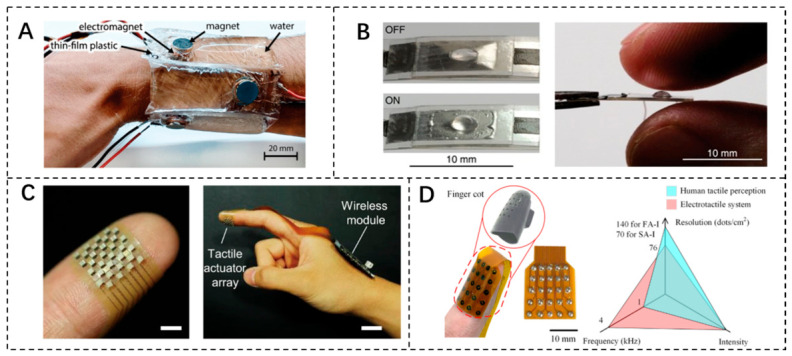
(**A**) The HAPSEA haptic feedback device, featuring flexibility and capabilities for squeezing, vibration, and localized force. Reprinted with permission from Ref. [103]. (Copyright © 2023, IEEE). (**B**) A hydraulic electrostatic actuator with a latency as low as 5 ms. Reprinted with permission from Ref. [105]. (Copyright © 2020 WILEY-VCH Verlag GmbH & Co. KGaA, Weinheim). (**C**) A piezoelectric actuator array with a spatial resolution of 1.8 mm; a wireless module on the back of the hand can drive the independent reproduction of haptic signals on each pixel. Reprinted from Ref. [107] under the terms of the CC BY license. (Copyright © 2022, The Authors). (**D**) An electrotactile feedback device at the fingertip. Compared to human tactile perception, this device exhibits a similar spatial resolution, a higher refresh rate, and covers the entire intensity range of human touch. Reprinted with permission from Ref. [108]. (Copyright © 2022, The American Association for the Advancement of Science).

Electromagnetic actuators rely on electromagnetic fields for energy conversion, offering advantages such as low driving voltage and large driving force [102]. For instance, Kohls et al. [103] proposed a soft electromagnetic actuator named HAPSEA for haptic applications, which further achieved a force output of 5.2 N under low voltage conditions (up to only 2 V) by leveraging hydraulic technology, while demonstrating excellent heat dissipation capabilities (with a surface temperature of only 36 °C), ensuring its safety in haptic applications (Figure 4A). However, such wearable haptic devices based on electromagnetic actuators frequently face challenges such as narrow working bandwidths and slow response speeds, which can easily cause interruptions or lags in the seamless interaction process, making it difficult to satisfy the real-time synchronization requirements of immersive haptic feedback [104].

In contrast, electrostatic actuators utilizing Coulomb’s law can overcome the slow-response limitations of electromagnetic actuators. For example, Leroy et al. [105] developed a hydraulically amplified electrostatic actuator with a response time of less than 5 ms, which can maintain an effective displacement output at a high frequency of 200 Hz (Figure 4B). This ultra-low latency effectively eliminates the temporal gap between user operations and system responses, serving as an important approach for achieving instantaneous, seamless haptic feedback. However, unlike electromagnetic actuators, electrostatic actuators typically require high-voltage driving, making them less easily integrated into small, portable haptic feedback devices than the former. This limits hardware integration and hinders the imperceptible wearing experience required for seamless interaction. Furthermore, electrostatic actuators are significantly affected by changes in environmental humidity or gas content, which can severely impact the effectiveness of haptic feedback [106]. Consequently, encapsulation design should be thoroughly considered in future studies to mitigate interference from the external environment.

With advancements in materials science, flexible piezoelectric materials can not only achieve efficient conversion between mechanical and electrical energy but also maintain excellent performance under bending and stretching. This flexibility allows devices to naturally conform to human skin, serving as a biomimetic interactive interface [109]. Benefiting from this, piezoelectric actuators not only exhibit outstanding performance in terms of response speed, driving force, working bandwidth, and miniaturization capability, but also hold immense potential in achieving high-definition tactile sensations [102]. These attributes make piezoelectric actuators widely regarded as the most suitable candidates for haptic feedback. For example, Jin et al. [107] developed an ultra-flexible, high-resolution wireless haptic interface system using piezoelectric actuator arrays. It achieves a spatial resolution of 1.8 mm and can precisely deliver tactile stimuli to the skin surface over a wide frequency range from 1 Hz to 1 kHz, mimicking the sensory dimensions and sensitive frequency bands of human biological mechanoreceptors (Figure 4C). Furthermore, it achieves micrometer-scale displacement control and clear texture simulation with an ultra-low latency of less than 1.55 ms. This high-resolution and untethered design physically eliminates bulky constraints, bringing the system closer to a truly imperceptible and seamlessly integrated artificial skin.

Comprehensively, compared to other electromechanical actuators, piezoelectric actuators offer greater advantages in terms of haptic feedback intensity, response controllability, and environmental adaptability. Table 3 displays a comparison among several haptic feedback devices based on different types of actuators.

##### Electrotactile Devices

Electrotactile devices are typically composed of electrodes and stimulators, which directly apply controlled electrical stimulation to the nerve endings of afferent nerves beneath the skin to activate surrounding mechanoreceptors, thereby eliciting tactile sensations. This process is not mediated by any skin receptor and involves no energy conversion, enabling tactile generation with virtually zero latency [112]. Because electrotactile devices do not rely on mechanical structures to generate feedback, they possess significant advantages in high-density integration; meanwhile, since the switching of electrical signals is far faster than mechanical motion, electrotactile devices typically achieve higher spatial resolutions and refresh rates [113]. For instance, Lin et al. [108] utilized a current-steering super-resolution stimulation technique to develop a wearable electrotactile rendering system with a high spatial resolution (76 dots/cm^2^) and a high refresh rate (4 kHz) (Figure 4D).

Furthermore, because the electrode and stimulator arrays themselves are exceptionally thin and light, they can be easily integrated into textiles such as gloves [114] and wristbands [115] when combined with flexible electronics technology, laying the physical foundation for imperceptible interaction. However, maintaining continuous haptic feedback is typically accompanied by frequent electrical discharges, which impose immense power consumption pressure; meanwhile, interactive interruptions triggered by battery depletion or frequent charging can severely hinder the seamless experience. To address this issue, Xu et al. [114] developed self-powered electrotactile textile haptic gloves, which achieve electrical stimulation through the triboelectric effect and gas breakdown discharge mechanisms. Tests demonstrated that users can generate sufficient mechanical energy to power the gloves solely through daily hand movements, eliminating the reliance on batteries or external power sources. This battery-free, self-sustained flexible textile design ensures enduring and continuous closed-loop haptic feedback without requiring manual charging interventions.

However, electrotactile feedback also confronts the challenges of inconsistent tactile sensations and potential pain risks caused by interface impedance fluctuations (such as sweating or electrode peeling). To this end, the system must incorporate precise modulation and safety mechanisms. Common countermeasures include mathematically modeling skin-electrode interface impedance changes to construct closed-loop regulation mechanisms [116], or employing voltage control techniques such as dual-channel high-frequency alternating current (AC) modulation to regulate the stimulation voltage [108]. Despite facing various difficulties in specific applications, electrotactile feedback remains a crucial technological direction for constructing lightweight, seamless wearable haptic interfaces due to its favorable energy efficiency characteristics and high miniaturization capability.

##### Thermo-Haptic Devices

Thermal haptic devices typically employ Joule heaters or thermoelectric actuators as their means of thermal generation, stimulating skin thermoreceptors through the controllable regulation of skin surface temperature to elicit tactile perceptions associated with cold, warmth, and temperature fluctuations [117]. From the perspective of system-level bionics, the human skin’s perception of the objective world in nature is inherently multimodal and profound. Although electrical stimulation and electromechanical actuators can effectively simulate the mechanical properties of objects (such as hardness and texture), the critical experience of material warmth/coolness and thermal transients in human tactile perception cannot be delivered by simple mechanical stimuli. By reconstructing thermal sensations to characterize virtual items, thermal haptic devices can provide users with a unique tactile experience of virtual objects, which is of paramount significance for enriching the sensory richness in seamless HCI.

Lee et al. [118] developed a soft wearable thermotouch haptic interface (T2HI) capable of simultaneously providing users with tactile and thermal feedback. Through user testing in a VR environment, they confirmed that compared to single-modal feedback, this combination significantly enhances the user’s perceived realism of virtual objects, effectively deepening the sense of seamless integration between humans and virtual environments. Nevertheless, this work also highlights a significant challenge for thermal haptic devices in practical applications: controlling heat accumulation to deliver an optimal thermal haptic experience to users without causing thermal burns. In addition to leveraging passive heat dissipation via natural processes such as evaporation, thermal conduction, convection, and radiation [119], developing real-time feedback algorithms to regulate the electrical current of Peltier devices for precise active cooling represents another viable approach [120]. This facilitates maintaining the dynamic thermal physiological homeostasis at the device-skin physical interface.

Furthermore, while most current research focuses on enabling users to accurately perceive the temperature of virtual objects, simply emphasizing temperature cannot perfectly reconstruct the thermal sensations associated with touching real objects, as it often overlooks the complex interplay between object thermal properties and human thermophysiology. Delving into the fundamental mechanisms of biological heat transfer, Woo et al. [121] proposed a thermal haptic feedback system based on a biothermal model. By simultaneously modulating skin temperature and heat flux, the system successfully differentiated materials with the same surface temperature but distinct thermophysical properties (e.g., wood and copper), keeping simulation errors under 1.2% for temperature and 6.6% for heat flux. This interactive design, based on the human body’s own physiological responses, enables users to attain a natural perception of the physical world without incurring an additional cognitive load, thereby driving the evolution of wearable closed-loop systems toward becoming “external organs” of the human body.

**Figure 5:**
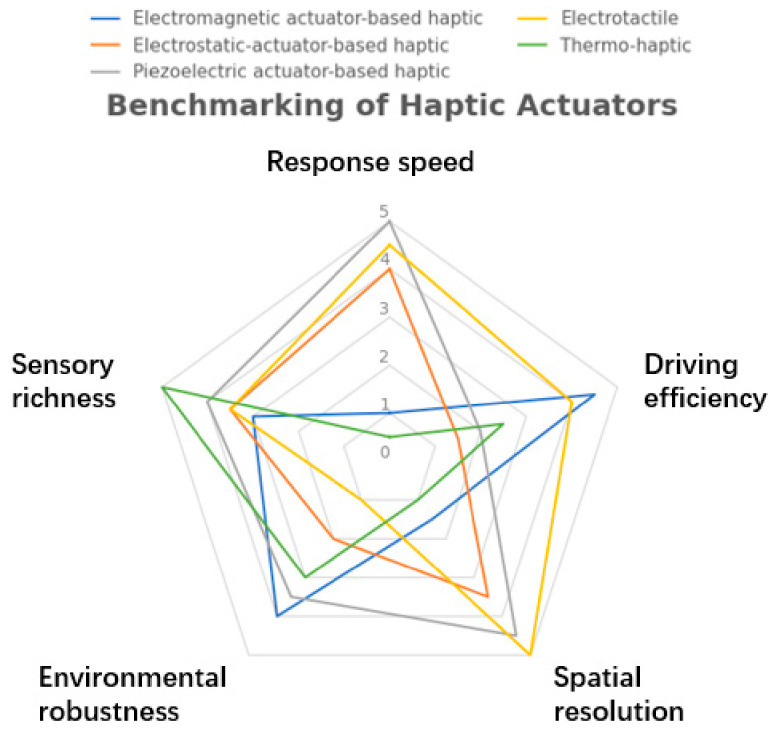
Visualized comparison of major haptic feedback technologies for Seamless HCI. The scoring (0–5) represents a relative qualitative assessment, where 0 indicates low performance or suitability and 5 indicates excellent performance or high suitability in each dimension.

To provide a comprehensive and critical evaluation of the discussed seamless HCI haptic feedback modalities, Figure 5 visually synthesizes a performance comparison across several key engineering and perceptual dimensions. Each technology exhibits unique trade-offs that determine its suitability for specific biomimetic interaction paradigms.

#### 3.1.2. Auditory Feedback

In seamless HCI, the unique advantage of auditory feedback lies in its biomimicry of the human capacity for subconscious omnidirectional perception of the surrounding environment. Unlike visual interaction, which demands highly focused attention, human audition can continuously process environmental information in the background without interrupting current physical tasks. Based on this principle, wearable auditory interfaces not only provide users with implicit, hands-free status feedback but also effectively mitigate the cognitive load in high-complexity tasks, serving as a crucial component in forging natural interactive experiences.

To achieve deep seamless integration, auditory feedback has transitioned from simple monophonic prompts to leveraging 3D spatial audio to reconstruct the user’s environmental perception. In vision-constrained application scenarios, the system can simulate the human binaural effect and spatial localization auditory mechanisms, converting obstacle orientation and distance data extracted by computer vision into continuous variations in sound intensity and phase differences between the left and right channels [25]. This data sonification strategy intuitively establishes a physical spatial mapping in the user’s mind without requiring deliberate attention allocation. This is essentially a biomimetic enhancement of human spatial perception mechanisms, effectively elevating the immersion and naturalness of the interaction.

Furthermore, to prevent auditory fatigue caused by overloading a single sensory channel, audition often requires biomimetic synergy with other senses (such as haptics) to achieve the intelligent offloading of multimodal information. For instance, in wearable navigation systems for visually impaired individuals, complex semantic information (such as global path planning and navigation instructions) is primarily conveyed via the auditory channel, whereas physical spatial interventions requiring immediate reflexes (such as close-range emergency obstacle avoidance) are assigned to the haptic channel [27]. This perceptual offloading mechanism highly aligns with the task allocation logic of the human central nervous system: delegating high-level cognitive tasks to the auditory semantic network while executing low-level reflexive actions through the haptic motor network, thereby achieving efficient and seamless human–computer collaboration without occluding any single sensory modality.

However, in open real-world environments, complex background noise can easily disrupt the continuity of auditory interaction. Here, the introduction of active noise control (ANC) technology is not merely to “enhance sound fidelity” but to maintain the reliability of the human–computer communication channel in noisy environments without increasing the cognitive load. Traditional filtering algorithms often confront performance bottlenecks when dealing with non-stationary real-world mechanical noise and may even introduce artifacts [122]. To address this, emerging research integrates deep learning (such as CNNs [123] or WaveNet architectures [124]) into ANC systems, leveraging the algorithms’ powerful capability to extract non-linear time-frequency features to precisely separate target interaction commands from background noise. For example, the generative fixed-filter active noise control (GFANC) architecture developed by Luo et al. [123] combines a lightweight CNNs with an underlying controller to achieve zero-latency noise reduction. Furthermore, when confronting environmental changes, the system can rapidly adapt by merely updating sub-filters rather than retraining the entire model, exhibiting excellent environmental transferability. Under computational and power constraints, this highly efficient AI-driven adaptive noise cancellation mechanism guarantees the seamless invisibility of the auditory feedback device as an “external organ” in all-weather HCI.

#### 3.1.3. Olfactory Feedback

As the only sensory modality among the human five senses that directly projects to the cerebral cortex without passing through the thalamus, olfaction perceives volatile chemical substances through receptors in the nasal cavity to identify odors. It plays a fundamental role in hazard warning (such as smoke and putrid odor recognition) and the shaping of social behavior, while possessing an irreplaceable and unique value in emotional regulation and deep memory evocation [125]. More importantly, olfactory perception typically does not require active attention allocation from users, enabling it to provide information feedback under an extremely low cognitive load, making it an ideal biomimetic channel for constructing implicit and seamless HCI environments.

To satisfy the requirements of seamless HCI for device unobtrusiveness and wearing comfort, odor generation technology in olfactory feedback devices has progressively shifted in recent years from mechanical approaches (such as transporting odors via micro gas pumps) to non-mechanical approaches (such as utilizing heating to trigger phase transitions of fragrances for release). This shift has enhanced the control precision of odor release, rendered the delivery process quieter, and minimized system volume, thereby providing the underlying hardware foundation for integrating olfactory feedback modules into compact, flexible wearable platforms [126,127].

By controlling the timing, duration, and combination of released odors, olfactory feedback can synergistically collaborate with other sensory feedbacks to create richer multimodal feedback. In particular, skin-integrated miniaturized odor generator (OG) arrays have been proven to significantly enhance presence and immersion in VR and AR applications. Flexible wearable olfactory interfaces developed based on these arrays have also demonstrated remarkable potential in 4D cinema and clinical olfactory training, achieving experiences unattainable by previous systems that relied solely on vision and audition [128]. Furthermore, psychophysiological studies have confirmed that introducing context-matching olfactory stimuli into VR environments can significantly enhance users’ emotional resonance and multi-organ immersive experiences from a neurophysiological level [129,130].

However, compared to haptic and auditory feedback, relevant research on olfactory feedback remains scarce. This is primarily because odor generation devices have underperformed in terms of miniaturization, simplification, and wearing comfort for a relatively long period [128], and the substantial latency between odor generation and its propagation to the user’s nasal cavity fails to satisfy the immediacy requirements of seamless interaction [131]. Therefore, next-generation olfactory feedback technologies must be optimized for miniaturization and low latency to facilitate better integration into wearable devices. Liu et al. [131] developed a smart olfactory interface featuring low power consumption, small volume, and high array density, incorporating a proprietary AI algorithm. By analyzing environmental data and user positioning to predict user actions, the system pre-emptively triggers odor release, achieving an olfactory feedback response time as low as 70 ms. Notably, the introduction of AI provides an active compensation mechanism for olfactory feedback, making up for the deficiencies of inherent physiological latencies in the olfactory channel (such as the olfactory adaptation process). This improvement in sensory synchronization is essentially a biomimetic simulation of the human proactive perception mechanism. By diminishing the lag between device response and physiological perception on a temporal scale, the system supports wearable devices in operating as “external organs” for invisible, seamless interaction with users from the underlying logic.

Since olfaction is intrinsically generated by the brain being stimulated by electrical signals produced when odor molecules bind to olfactory receptors inside the nasal cavity, some novel studies have recently attempted to bypass odor molecules entirely and directly utilize electrical stimulation to evoke olfaction, aiming to construct a true zero-latency seamless closed-loop interaction. However, although some non-invasive electrical stimulation studies have demonstrated the feasibility of directly eliciting olfaction via electrical stimulation and achieving sensory lateralization [132], the elicitation efficiency remains low. Furthermore, these methods confront the challenge of precisely controlling the electrical stimulation zone without cross-stimulating adjacent nerves, which places this technology some distance away from practical deployment [133]. Future optimizations could consider methods to achieve precise stimulation of the olfactory receptor distribution areas, as well as incorporating AI for real-time control over the intensity and frequency of the electrical current, thereby ensuring safe and efficient guidance of olfactory elicitation.

### 3.2. Actuation-Based Feedback

Distinct from sensory feedback that focuses on transmitting status information to the user, executive feedback emphasizes that after perceiving changes in human states, the device actively exerts controllable physical or physiological interventions acting directly on the human body, thereby constructing a “perception-decision-intervention” closed loop. This type of feedback mechanism exhibits profound biomimetic value in the maintenance of physiological homeostasis; by drawing inspiration from the working mechanism of the human pancreas, a biomimetic artificial pancreas integrating continuous glucose monitoring and automated infusion pumps can achieve real-time closed-loop regulation of blood glucose concentrations within the body’s internal environment. This unconscious interaction mode maintains organismal homeostasis without requiring subjective user intervention [134]. Although this implicit interaction holds an irreplaceable position in constructing an unconscious, seamless interactive experience, to explore the dynamic enhancement characteristics in human–computer collaboration in a more targeted manner, this section will focus on outlining the implementation modalities and applications of executive feedback within two critical pathways: musculoskeletal assistance and biomimetic neural interfaces.

#### 3.2.1. Biomimetic Musculoskeletal Interfaces for Mechanical Feedback

Through exoskeletons—locomotion assistive devices attached to the human body surface—wearable systems can utilize external mechanical mechanisms to apply physical torques to human limbs or joints, providing mechanical feedback at a macroscopic level to assist or augment human locomotion capabilities in certain scenarios. Rigid exoskeletons offer the advantage of high torque output, but they are often bulky and prone to misalignment with the natural motion trajectories of the human body [135]. Therefore, current exoskeleton designs are gradually incorporating biomimetic design philosophies. They are constructed using advanced hardware such as textiles, pneumatic artificial muscles, or flexible cable drives, while leveraging real-time motion recognition technologies to closely conform to human body contours, dynamically adapt to complex motion trajectories, and target the adjustment of the magnitude and direction of the torques applied to the user. This design approach mimics the mechanical properties of actual muscles and tendons, allowing compliant physical interfaces to better adapt to complex movements while ensuring wearing comfort, thereby providing a hardware foundation for achieving seamless physical interaction [136,137].

However, relying solely on hardware compliance is insufficient to achieve deep seamless interaction. If the control strategy of the device lags behind the user’s true movement intentions or generates misjudgments, it will still cause the device to apply opposing resistance to the human body. Consequently, accurately decoding motor intentions is key to improving system interaction compliance. Because physiological signals characterizing motor intent, such as sEMG, exhibit pronounced non-linearity and individual variability, some studies have introduced deep learning networks to enhance the robustness of intent decoding. For instance, some research has combined generative adversarial networks (GANs) with graph attention mechanisms to effectively extract the spatial and temporal features of sEMG signals under small-sample conditions [28]. Based on this highly robust intent decoding, the following controller of the exoskeleton can more accurately regulate the output torque, thereby reducing the disagreement in motor intent between the human and the machine.

Furthermore, for exoskeletons to truly function as external organs of the human body, system latency must mimic human neural conduction speeds; that is, the assistive torque output by the device should be synchronized with the human motor intent. To this end, predictive mechanisms need to be introduced to compensate for latency deficiencies. For example, Carvalho et al. [138] utilized LSTMs to process time-series data from wearable sensors, enabling the system to recognize locomotion mode transitions in advance (such as transitioning from level ground walking to ramp climbing) with a lead time of approximately 0.66 s, and to pre-set the corresponding assistive torque. This shift in control strategy from reactive response to proactive prediction further elevates the degree of seamlessness of locomotion assistance systems in HCI. However, this improvement in predictive accuracy is, in many cases, built upon a compromise regarding computational power requirements, which may make high-precision models difficult to deploy on computation-constrained edge devices.

Through the integrated optimization of physical interfaces, intent decoding, and system latency, exoskeletons can already serve as an extension of the human body, interacting seamlessly with the user. To provide a more intuitive presentation of the biomimetic locomotion assistance schemes currently developed for different anatomical sites, Table 4 compares representative soft exoskeleton systems for several body parts.

#### 3.2.2. Biomimetic Neural Interfacing and Closed-Loop Feedback

Direct neural interaction utilizes the universal communication medium within living organisms—action potentials (electrical signals)—to establish the most natural communication bridge between devices and the human body through real-time, bidirectional information transmission with the central or peripheral nervous system in a physiologically compatible manner. This neural interface-based interaction paradigm enables wearable devices to bypass general sensory pathways, significantly enhancing the efficiency and depth of feedback, making it a crucial pathway for achieving seamless HCI [144].

In practical dynamic interaction scenarios, the core of a seamless closed loop lies in the adaptive maintenance of dynamic homeostasis. To address the issue where open-loop transcutaneous electrical nerve stimulation (TENS) is prone to inducing biological neural adaptation that leads to feedback failure, Du et al. [29] introduced a capability to capture sEMG variation characteristics in real time into a wearable TENS platform. By utilizing sEMG as the input signal, the system dynamically adjusts neural electrical stimulation parameters with a latency of less than 10 ms. This closed-loop mechanism achieves adaptive, bidirectional interaction between the user and the device. Notably, with breakthroughs in hardware miniaturization technology, this bidirectional interaction has extended to the central nervous system level, supporting synchronous recording and high-fidelity closed-loop neuromodulation of single-neuron activity in freely behaving states [145]. This precise, bidirectional interaction at the microscopic scale allows wearable devices to integrate with the nervous system on both physical and informational levels, laying a biomimetic foundation for constructing the next generation of intelligent neural prostheses and deeply immersive human–computer collaboration platforms.

Furthermore, by seamlessly integrating neural stimulators with prostheses or exoskeletons, wearable devices can convert pressure and texture information captured by mechanical sensors into biomimetic neural electrical pulse trains, reintroducing them into the residual nerves of amputees [146]. This neural feedback mechanism, capable of seamlessly restoring genuine proprioception, not only allows patients to reacquire authentic perceptions of the external world through their impaired limbs but also provides empirical insights for locomotion assistive devices designed for healthy users, marking a critical milestone in the evolution of wearable devices from mere tools to biomimetic external organs.

## 4. Challenges and Future Perspectives

In summary, this paper comprehensively reviews the recent advancements in seamless HCI driven by flexible biological interfaces and intelligent systems. From bridging the physical modulus gap using biomimetic flexible materials (such as hydrogels, MXenes, and paper-based electronics) to acquire high-fidelity signals, to relying on ML and edge computing to achieve the precise decoding of complex intentions and cross-user generalization, and further to closed-loop execution frameworks integrating multisensory (haptic, auditory, and olfactory) feedback and physical interventions, wearable devices are accelerating their evolution toward becoming “external organs” of the human body. However, despite the substantial progress achieved at the current laboratory proof-of-concept stage, pushing these systems toward all-weather, highly robust real-world applications still faces non-negligible limitations in underlying materials, system integration, feedback architectures, and algorithmic ethics. Future research urgently needs to achieve paradigm breakthroughs in the following dimensions.

### 4.1. Material Durability and Environmental Stability

Although hydrogel- and MXene-based sensors possess excellent skin conformability in their initial states, they inevitably face microstructural fatigue, polymer chain breakage, and non-linear electrical hysteresis caused by viscoelasticity under high-frequency, large-strain, repeated mechanical loading in the real world. This intrinsic degradation, compounded by severe motion artifacts, leads to the drift of interactive signals. Furthermore, although highly biocompatible substrates represented by paper-based materials exhibit excellent biomimetic breathability, these hydrophilic porous networks are highly prone to structural softening and interfacial delamination when exposed to extensive sweat infiltration or high-humidity environments. Future material designs must transcend the mere scope of “flexibility” and evolve toward “environmental adaptability.” By introducing multiple dynamic reversible chemical bonds and self-healing networks at the molecular level, or by constructing asymmetric encapsulation films with anisotropic moisture-resistant and breathable properties, the absolute stability of electrical performance can be maintained amidst extreme environmental fluctuations, thereby providing a temporally coherent underlying data stream for seamless interaction.

### 4.2. Internal Latency in Multimodal Feedback

Although multimodal sensory rendering and executive physical feedback have achieved remarkable success in reshaping HCI immersion and locomotion assistance, system latency remains the core engineering bottleneck hindering a deep seamless experience. Whether due to the inherent physical response times of the hardware (such as the slow diffusion of olfactory odor molecules or the heating process of thermal haptics) or the computational lag brought about by traditional long “edge–center–edge” communication links, both cause the device’s interventions to lag behind the user’s actual state, even triggering dangerous human–machine torque confrontations during locomotion assistance. For sensory feedback with high error tolerance and slow macroscopic movements, leveraging AI to construct proactive compensation is an effective pathway to overcome physical latency; examples include utilizing edge AI algorithms to control the pre-emptive release of olfactory feedback odors and employing LSTMs to accomplish exoskeleton torque pre-setting. However, for high-frequency physical interventions requiring instantaneous responses (such as neuromodulation or delicate musculoskeletal synergy), relying solely on central AI computation still introduces non-negligible communication bandwidth and computational power burdens. Future interactive interfaces should utilize threshold-switching memristors [147] or optoelectronic synaptic transistors [148] to construct localized neuromorphic closed loops. This architecture allows external stimuli or faint physiological commands to directly complete asynchronous pulse decoding locally at the sensor nodes, triggering actuator actions in situ without the intervention of the main CPU. Combined with neuro-inspired transparent interaction strategies [149], this innovation in the underlying architecture is expected to fundamentally compensate for computational latency, achieving true microsecond-level, low-power seamless human–computer synergy.

### 4.3. System Integration and Power Supply

Existing interactive closed loops mostly rely on the heterogeneous stacking of discrete components, wherein flexible front-end interfaces, micro-power supply devices (such as batteries or triboelectric nanogenerators), and rigid back-end wireless communication and processing circuits are interconnected via physical wires. This mechanical mismatch at the rigid-flexible junctions not only elevates interfacial impedance but also creates vulnerable points of stress concentration during limb movements, ultimately leading to system failure. In addition, high-density sensory feedback (such as tactile and thermal rendering) induces immense instantaneous power consumption, imposing severe endurance pressures on current flexible power modules.

Leveraging monolithic integration technology, future HCI systems will be capable of achieving the in situ, seamless integration of “perception–computation–power supply-wireless communication” on a single flexible substrate. This system-level integration strategy can fundamentally eliminate the stress concentration issues at the interfaces between rigid chips and flexible substrates in current hybrid systems. By overcoming mechanical impedance mismatch, it enables conformal contact with biological tissues [144]. This not only maximizes energy density and signal transmission efficiency at the physical level but also allows the device morphology to infinitely approximate the actual biological epidermis, serving as the ultimate hardware foundation for next-generation seamless HCI.

### 4.4. Algorithm Trustworthiness in Wearable HCI Systems

At present, the remarkable performance of deep learning models is predominantly built upon static, controlled, and homogeneous lab-controlled datasets. When confronting uncontrollable domain shifts in long-term, real-world scenarios, their intention-decoding accuracy often deteriorates sharply. Simultaneously, in applications involving physical safety (such as locomotion assistance), black-box decisions lacking transparent logic can easily introduce unpredictable medical risks. Furthermore, the continuous uploading of multimodal physiological features raises highly sensitive data privacy concerns. Therefore, next-generation systems must construct trustworthy AI.

On the one hand, explainable artificial intelligence (XAI, such as attention mechanisms and SHAP) must be comprehensively introduced to visualize the decision weights of black-box networks across spatiotemporal dimensions, allowing them to undergo cross-validation by human physiological prior knowledge and establishing a safety baseline for physical applications. On the other hand, concerning data security, future algorithmic deployment should rely on federated learning and differential privacy technologies, in tandem with highly generalizable wearable foundation models. Through a cryptographic, distributed framework based on “keeping data local while aggregating parameters with noise,” precise and secure personalized seamless interaction can be realized while fully safeguarding individual biometric privacy.

## Figures and Tables

**Table 1:** Comparison of representative work based on MXene, hydrogel and paper-based materials. In this table, GF stands for gauge factor.

Materials	Application	Sensitivity	Stretchability	Durability	Response Time	Ref.
MXene	Pressure sensing	334.1 kPa^−1^ 0–150 kPa detection range	80% strain (140 kPa compressive stress)	Stable after >4000 cycles (3 kPa pressure)	0.29 s for response 0.20 s for recovery	[41]
	Pressure-temperature sensing	35.7 kPa^−1^ 6.5 Pa detection limit 0–20 kPa detection range	42.38 MPa tensile strengeth	Stable after >4400 cycles (5 kPa pressure, 8000 s)	0.25 s for response 0.20 s for recovery (1 kPa pressure)	[44]
	Strain sensing	0.05% detection limit 933 GF (180–240% strain)	240% strain	Stable after 33,000 cycles (20% strain)	200 ms	[52]
Hydrogels	Strain sensing	0.7 GF (0–300% strain) 1.5 GF (300–600% strain) 2.3 GF (600–866% strain)	>10,000% (original) 860% (packaged strain gauges)	Stable after >1000 cycles (electrical hysteresis < 1%)	238 ms (5% strain, 500 mm/min loading and uploading rate) 0.12 s self-healing time	[45]
	Curvature sensing	1.97 µW m^−1^ (light intensity) 0.04 nm m^−1^ (light wavelength) 0–139.84 m^−1^ detection range	/	Slight fluctuations in output light intensity after 200 times	/	[34]
	Strain sensing	1.98 GF (0–80% strain) 3.28 GF (120–160% strain) 0–160% sensing range 0.1% detection limit	160% strain 27 kPa shear strength 130 kPa tensile strength	Stable after 800 cycles (original sensor, 20% strain) Slight decrease in detected peak value after 200 cycles (healed sensor, 20% strain)	257 ms (original, 10% strain) 317 ms (healed, 10% strain)	[35]
Paper-based materials	pH sensing	46.01 mV pH^−1^ 1.53–13.65 pH detection range (steps of 0.5)	/	0.01 mV/h signal drift after 12 h Stable after 5000 bending cycles	/	[37]
	Humidity sensing	Excellent linear response of capacitance change (65.3–97.6% RH) 6.4–97.6% of RH detection range	5–25% biaxial strain	Stable after 1000 stretching cycles (5–25% strain)	155 s from 6.4% to 90% RH 58 s from 90% to 6.4% RH	[49]

**Table 2:** A summary of commonly used ML algorithm in wearables.

Algorithm	Suitable Signals	Typical HCI Tasks	Core Matching Rationale	Ref.
Support vector machines (SVM)	Sparse-channel sEMG; Feature-extracted EEG signals for BCI	Few-shot personalized calibration; discrete motor intention classification (e.g., binary gesture decoding)	Maximizes the classification margin hyperplane to prevent overfitting in small-sample and high-dimensional spaces. Highly robust for rapid, on-device personalized calibration using minimal initial data.	[53]
Random forest (RF)	Multimodal heterogeneous signals (e.g., fusing IMU kinematics with ECG/PPG or temperature metrics)	Multimodal Human Activity Recognition (HAR); continuous health tracking	Naturally resists noisy or missing data and handles heterogeneous features without complex normalization. Tree-based logic provides high “white-box” interpretability.	[54]
K-nearest neighbors (k-NN)	Low-dimensional extracted features (e.g., statistical features from IMU or simple impedance changes)	Simple static posture classification; on-device dynamic incremental learning	Operates via a “lazy learning” mechanism with no explicit training phase. Calculates distances to stored prototypes for high efficiency in on-device incremental updates and continuous personalization.	[55]
Convolutional neural networks (CNNs)	High-density sEMG arrays signals; multi-channel tactile/flexible sensor grids	Complex hand gesture recognition; spatial anomaly detection; fine motor decoding	Naturally extracts high-dimensional spatial topological patterns through local receptive fields and weight sharing. Inherent translation invariance for robustness against electrode displacement, sensor shifts, and skin deformation movements.	[56]
Long short-term memory networks (LSTM)	Continuous time-series data streams (e.g., continuous IMU kinematics or dynamic force sensors)	Continuous exoskeleton gait prediction; dynamic motion intention forecasting; sequential physiological trend tracking	Utilizes internal gating mechanisms to capture and retain long-term temporal dependencies in cyclic or continuous data streams. Predicts future states based on past temporal contexts to reduce latency in locomotion assistance	[57]

**Table 3:** Comparison between tactile feedback devices based on different actuators.

Actuator	Driving Voltage	Driving Efficiency	Response Time	Power Use	Spatial Resolution	Ref.
Electrostatic actuators	Up to 1400 V	0.3 N force 500 μm out-of-plane displacements 760 μm lateral motion	<5 ms (100 W/kg specific power)	/	<10 mm actuator design spacing	[105]
Electrostatic actuators	Up to 6000 V	2.44 mm actuation stroke >2.3 N controllable force	/	3.0 mW when holding an extended position	/	[110]
Electromagnetic actuators	2 V	Up to 5.2 N 0.4 mm stroke length 10 μm vibration displacement @200 Hz	/	1.3 W (continuous operation) 0.056% electromechanical efficiency	/	[103]
Electromagnetic actuators	≤7 V (max force output)	0.4 N @ 7 V 0.63 mm resonance displacement @ 0.1 Hz	44.6 ms for reaching 90% max fore output	/	/	[111]
piezoelectric actuators	40–60 Vpp	2.1 m/s^2^ acceleration @ 60 Vpp (21 times of human vibrotactile threshold) 1.3 μm displacement @ 60 Vpp (8.5 times of human pacinian threshold)	<1.55 ms	/	1.8 mm pitch static pressure sensor 4.8 mm pitch dynamic pressure sensor	[107]

**Table 4:** Comparison of exoskeletons used in different locations.

Target Area	Intention Decoding & Control Strategy	Strength Augmentation	Real-World Application	Ref.
Hand	Geometric point cloud analysis for adaptive visual servoing control without EMG	91 ± 2% grasp ability score	Adaptive grabbing for unknown complex geometric objects	[139]
Hand	Hybrid rigid-soft mechanism using differential transmission for force distribution	Exerted force up to 16.78 N	Improve wearability and comfort in daily interactive activities	[140]
Upper limb	KNN algorithm for motion recognition and Fuzzy PID for active control	15% weight reduction (compare to traditional rigid exoskeletons)	Upper limb movement assistance in work settings or rehabilitation	[141]
Spine/lumbar	Spine-inspired passive compliance mechanism adapting to user’s trunk posture	12 N·m extension moment at the L5-S1 joint 20–30% reduction in erector spinae activity in stoop lifting	Daily stoop lifting and squat movement assistance	[142]
Hip	IMU-based LMR adjusting assistance for different terrains	10.5 ± 2.3% and 12.1% metabolic cost reduction in level walking and stair ascent	Adaptive assistance for walking on flat ground and going up and down stairs	[137]
Anklebone	Human–machine torque confrontation control combined with neuromusculoskeletal model	5–7 N·m assistive torque	Gait abnormality correction and walking rehabilitation	[143]
Ankle-foot	LSTM neural network predicting locomotion modes using IMU and laser data	0.66 s gait patter prediction advance 98% prediction accuracy	Zero-delay mechanical propulsion in complex real-world environments (stairs, ramps, etc.)	[138]

## Data Availability

No new data were created or analyzed in this study. Data sharing is not applicable to this article.

## Abbreviations

The following abbreviations are used in this manuscript:

AI	Artificial intelligence
ANC	Active noise control
BCI	Brain–computer interfaces
BSS	Blind source separation
CNNs	Convolutional neural networks
EEG	Electroencephalogram
EMG	Electromyography
EOG	Electrooculogram
GANs	Generative adversarial networks
HAR	Human activity recognition
HCI	Human–computer interaction
HDC	Hyperdimensional computing
IMUs	Inertial measurement units
k-NN	K-nearest neighbors
KPCA	Kernel component analysis
LSTM	Long short-term memory
ML	Machine learning
MXenes	Transition metal carbides/nitrides
OGs	Odor generators
RF	Random forest
SOBI	Second-order blind identification
SVM	Support vector machines
TENS	Transcutaneous electrical nerve stimulation
2D	Two-dimensional
3D	Three=dimensional
